# How Workers Live—The Cotton Industry

**Published:** 1894-07-14

**Authors:** 


### Studies in Applied Sociology.

### XII.—HOW WORKERS LIVE—THE COTTON INDUSTRY.

In dealing with the cotton industry we have to do with a labour which is equally important in Europe and America, in Lancashire and Louisiana. Formerly these two names implied the raw and the finished product respectively, but protection is having its effect, and cotton manufacture is becoming an American industry also. While, therefore, we contrast the earnings of the skilled English operative with those of the cotton-picker of the South, we have also to take into account the States of Maine, New Hampshire, New York, and others where cotton factories are now established. These, indeed, form the more convenient bases of comparison. To make the comparison more just we must also take into account only the normal families; that is, those where the family consists of husband, wife, and not more than five children.

Comparing these normal families on either side of the Atlantic, we find that almost everywhere wives and children contribute to the family income. In Massachusetts the proportion of wives who work is large—one in four—and their average contribution to the family income is nearly half that of their husbands. Children are valuable aids to income in Massachusetts, they work in the proportion of one to three men, and their united contribution to the family income, in such families as are fortunate enough to have them (the average number of children of working age is 2·2) exceeds the father's earnings. The total income of these families averages about £145. The average total income of a similar family in Britain is about £115, the husband's earnings being here a little larger than the children's, and about twice that of the wife. In every district where the wife does not go out to work, and where the family is small, some additional income is obtained by keeping boarders or lodgers. Massachusetts is not, however, the State where the largest incomes are made at the cotton industry. Louisiana, New Hampshire, Pennsylvania, and New York all show a higher total income. In New York State the average income per family is about £165. It is not there, however, but in Rhode Island that one may expect to see the greatest amount of comfort. Small families are the fashion there—2·9 children per family, and a fifth of those from whom the statistics of the labour bureau were gathered had none at all. The result is that we find that the average income there is £25 per individual. Louisiana even exceeds this by £1 a head, though there the wives do not work, and childless families are almost unknown. But there the husband's income alone averages £133 per annum, and receipts from children and boarders bring the average family income up to £154. Happy Louisiana, where wages are high and the climate makes living cheap. But Louisiana "has it out" in house rent, for which the family pays £22 10s., and gets only a fraction over three rooms for that. Alas! Louisiana also spends more on intoxicants than any other State—something like £7 per family.

We have compared the earnings only of British cotton operatives with those of the United States, because they alone approach the American average. Georgia, South Carolina, and Virginia indeed show a comparatively low income; Virginia touches the lowest point at £93 10s. per family, but that is above the average family income of either France (£76), Switzerland (£74), or Germany (£63).

Having seen the amount of money earned, it follows next to find out how it is spent. It appears that the American generally spends more on rent than his European fellow; but he also goes in for more accommodation. The Englishman pays between £10 and £11 for a little over four rooms per family; the Swiss gets the same accommodation for half the money; neither French nor German aspire to quite three rooms, and pay respectively £7 and £5 16s. annually. Only in the State of Mississippi is the state of things comparable to this, and there the same accommodation as in Germany costs half as much again. But in all the Eastern States the house room amounts to five apartments or a fraction more, while for almost the same accommodation the rent varies from £10 in Connecticut to £19 in Pennsylvania. Louisiana we have already mentioned, and indeed throughout the Southern States poor accommodation and high prices seem to be the rule.

To deal with the expenditure on food is difficult, so differently do the various articles bulk in the consumption of different countries. We can therefore give only totals, without itemising the proportion of beef and bread that belongs to each. To do so, indeed, is impossible, when the Briton lumps all his animal food together as "meat," which the American analyses into beef, hog products, poultry, &c. We find, however, on the whole that the British cotton operative spends about £50 a year on the food of his family, and the cotton worker of Alabama exactly the same sum. The Germans live most cheaply of all, something under £30 representing the family expenditure for food; then come the French with £34, and the Swiss with £37. At the other end stands the worker of Connecticut, who averages £78 for food, while New Hampshire follows with £74 Louisiana with £67, and New York with £66.

With regard to clothing, we find the pleasing fact that the Englishman and his wife spend each just the same sum on dress, a little over £5, while £9 clothes the children. This is evidently the pet extravagance of the Britisher; for though clothes, especially men's clothes, are much dearer in the States than here, the average expenditure for men is nearly the same, while for women it is less, save in South Carolina, where Madame L'Americaine, who in this case is probably a lady of colour, expends 10s. more on her personal adornment. But on children's clothes much more is spent in America; £22 in New York, £17 in Pennsylvania, it falls to the British average only in poverty-stricken Mississippi. Of other expenditure, we find that the American spends four or five times as much on furniture as his English fellow-workman, about the same on books and newspapers, and very much less on amusements. In Pennsylvania the expenditure per family is barely £1, while in Britain it amounts to £7 10s. Only in Rhode Island, which in clothing, food, and fuel is the most economical of the Northern States, does the amusement bill run up to over £9. The average may be taken at £2. One other expenditure remains to be noted. On labour organisations the Englishman spends £2 10s. per annum; the workmen in Connecticut 30s.; in most of the other States not more than 25s., while in several, including Pennsylvania, no expenditure under this head is recorded

